# “I’m living in a ‘no’ world now…”- A qualitative study of the widespread impact of living with chronic breathlessness, and experiences of identification and assessment of this symptom in an older, frail community-based population

**DOI:** 10.1038/s41533-024-00409-3

**Published:** 2025-01-24

**Authors:** Helene L. Elliott-Button, Miriam J. Johnson, Ann Hutchinson, David C. Currow, Joseph Clark

**Affiliations:** 1 Wolfson Palliative Care Research Centre, Hull York Medical School, University of Hull, Hull, UK; 2 IMPACCT (Improving Palliative, Aged and Chronic Care through Clinical Research and Translation), Faculty of Health, University of Technology Sydney, Ultimo, NSW Australia

**Keywords:** Respiratory signs and symptoms, Geriatrics

## Abstract

Chronic breathlessness is a debilitating symptom with detrimental impact on individuals and carers. However, little is known about the experiences of community-dwelling, frail, older adults living with chronic breathlessness. To explore, (i) the psychological impact of living with chronic breathlessness, (older frail adult patients, carers) and (ii) how patients, carers, and clinicians experience identification and assessment of chronic breathlessness in the primary care setting. In-depth semi-structured interviews with eligible older adults (≥65 years; moderate to severe frailty [electronic Frailty Index >0.36]), and carers recruited from a community-based Integrated Care Centre in England. Clinicians were recruited from the Centre and affiliated GP practices. Recorded in-person interviews were transcribed and subjected to reflexive thematic analysis using Total Dyspnoea and Breathing Space conceptual frameworks. 20 patients (9 females), carers (4 spouses, 1 daughter), and clinicians (5 GPs, 3 advanced clinical practitioners, 2 nurses) were interviewed. Four themes were identified: (1) Widespread negative impact of chronic breathlessness. Breathlessness adversely impacts physical and psychological wellbeing. (2) Barriers to optimal health-seeking and identification of chronic breathlessness. Breathlessness is ‘one of many’ symptoms, and not prioritised in ‘one appointment, one problem’ consultations. Clinicians do not routinely ask about breathlessness. Patients are unaware of breathlessness-specific therapies. (3) Variations in chronic breathlessness management. Management is limited; few are offered evidence-based treatments (e.g., handheld fan) and patients find their own strategies. (4) Need for education and information. Clinicians felt helpless about breathlessness management, and patients lacked understanding and had low expectations of receiving help for this symptom. Breathlessness adversely impacts the psychological wellbeing of older frail adults. Chronic breathlessness in older, frail adults is invisible, unidentified and unmanaged in primary care. Evidence-based breathlessness interventions are available, but not routinely implemented with few patients accessing them. Proactive identification, assessment and management of breathlessness in primary care is needed to support adults living with chronic breathlessness.

## Introduction

Chronic breathlessness is a distressing symptom with major detrimental impacts on individuals, and their family and friends (carers)^1^. To avoid breathlessness, people often limit activities, entering a deconditioning cycle leading to poor mobility, fewer social interactions, anxiety, depression, and diminished quality of life^1–6^. Chronic breathlessness is also associated with higher health service utilisation^7,8^ and carers experience considerable negative physical, social, and emotional impacts^9–11^ as a result of providing care and undertaking extra responsibilities, often disregarding their own needs in the process^12^.

Chronic breathlessness is prevalent in the general population^13^, with about one in ten people living with breathlessness limiting exertion^14^. Breathlessness is more prevalent with older age^15^, cardiorespiratory diseases, and cancer^16,17^.

Effective, evidence-based interventions for breathlessness are available for use alongside disease-directed therapies^18–21^ but breathlessness is often not discussed during clinical consultations^22^ or seen as a therapeutic target. Such “invisibility”^22–24^ is a concern. To raise awareness of this problem, chronic breathlessness has been described as a distinct clinical entity^25^. Older adults may be particularly disadvantaged, especially if frail^26^ with accompanying sarcopenia^27^ which may further worsen breathlessness^28^. Older adults with or without frailty are more likely to have multiple long-term conditions many of which can cause breathlessness^29^.

Older adults with breathlessness are more likely to attend primary care services than those without^8,30^. However, little is known about the psychological impact of breathlessness or the experiences of breathlessness-related care within the primary care setting. Therefore, in a UK community setting, we aimed to explore i) the psychological impact of living with chronic breathlessness in frail older adults (patients, carers) and ii) how patients, carers, and clinicians experience identification and assessment of chronic breathlessness in this setting.

## Methods

### Design

In-depth semi-structured in-person interviews with patients, their carers, and clinicians were conducted. The study draws on aspects of interpretivism, considering that reality is subjective and constructed by the individual^31^. The study was embedded as part of the Proactive Anticipatory Care Evaluation (PACE study) of a new community-based integrated care service for older adults at risk of severe frailty (details published elsewhere)^32^. The project received NHS ethical approval (18/YH/0470; IRAS Project ID 250981) in accordance with the Declaration of Helsinki.

### Participants and recruitment

Eligible participants were community-dwelling older adults at risk of severe frailty (electronic Frailty Index >0.36 [moderate to severe frailty], ≥65 years) self-reporting breathlessness for at least 4 weeks, and their carers, attending a community-based Integrated Care Centre (ICC) in England^32^ between April and August 2019. Clinicians were recruited from the ICC and affiliated general practices (GP practices). Written, informed consent was gained prior to data collection.

### Sampling

Patients were purposively sampled according to sex and modified Medical Research Council breathlessness scale (mMRC) score (1/2 or 3/4). A convenience sample of carers and clinicians was recruited. A sample size of about 20 to 25 patients and carers, and 10 clinicians was anticipated to provide sufficient information power given the narrow focus of the topic^33^. Information power indicates that the more information within the sample – relevant to the study – the smaller the sample can be. Sufficient information power depends on study aims, sample specificity, use of theory, dialogue quality, and analysis strategy^33^. Recruitment ceased when it was agreed amongst the team that sufficient information had been obtained to answer our research questions.

### Data collection

Interviews were conducted face-to-face (except for three telephone interviews with clinicians) at a time and place convenient to the participant by a female PhD student in her mid-30s (HE), with a background in health psychology and experience of qualitative health research. Audio-recorded interviews (with a digital voice recorder) were conducted between 1st June 2019 and 31st March 2020.

Interviews used a topic guide based on team expertise, the literature, theoretical frameworks, and Patient and Public Involvement (PPI) review. Issues covered related to chronic breathlessness and psychological impact, quality of life, adaptations of activities, lived experience of interactions with primary care clinicians, views about legitimacy of breathlessness as a reason for consultation, and views about chronic breathlessness terminology. See appendices A and B (respectively) for patient/carer and clinician topic guides.

### Data analysis

Anonymised, verbatim transcripts were subjected to reflexive thematic analysis using Braun and Clarke’s six-step typology^34,35^. Following familiarisation with the transcripts, two researchers (HE and AH) independently conducted line-by-line coding of two transcripts inductively (codes rooted in the data) and deductively (codes mapped onto pre-existing frameworks of Total Dyspnoea^36^ and Breathing Space^1^), and then agreed a coding framework. This helps minimise bias and increase credibility. The remaining transcripts were then coded by HE using the coding framework. HE then grouped codes into preliminary descriptive themes which were then discussed in-depth and refined into analytic themes with other team members (JC and MJ). Data were managed using NVivo 11. Illustrative quotes are presented to provide detailed participant descriptions. Rigour of the analysis can be considered using Lincoln and Guba criteria^37^. ‘Credibility’ is demonstrated by use of two researchers to conduct coding; ‘Transferability’ is demonstrated by use of ‘detailed participant accounts’ which could allow the reader to determine whether these findings are transferable to their own research; ‘Dependability’ is demonstrated through specific step by step accounts of the analytic process; and ‘Confirmability’ is demonstrated through results being firmly rooted in the data.

### Theoretical frameworks

The Total Dyspnea^36^ framework aims to understand the physical, psychological, social, and existential (spiritual) experiences of breathlessness. This was developed similarly to Total Pain, an original concept established by Dame Cicely Saunders^38^. The Breathing Space^1^ framework describes the widespread effects of breathlessness by considering the impact of patients’ coping (engaged or disengaged), help-seeking behaviours (for persistent breathlessness or in crisis only), and clinicians’ responsiveness to breathlessness (clinician responsive to breathlessness and underlying disease *or* clinician responsive to underlying disease only).

These frameworks were applied during data analysis by having the frameworks at hand as coding/theme development occurred, to allow codes to be mapped onto relevant domains.

In the results section, relevant quotes have been given a descriptor to show which theoretical framework and domain they belong to (if applicable).

## Results

In total, 35 participants were interviewed (20 patients, 5 carers, lasting between 17 and 140 min; 10 clinicians lasting between 26 and 55 min). Most patients reported experiencing long-term chronic breathlessness (several years). Most reported worsening of breathlessness over time.

Participant characteristics are summarised in Tables 1 and 2.

**Table 1:** Participant Characteristics (Patients, carers).

Participant Characteristic	Total patient population, n = 20
Sex, n	
Female	9
Male	11
Age (years)	
Median (IQR)	78.5 (71.5–83.0)
Range	68–92
mMRC Scores	
1/2^a^	10
3/4^a^	10
Number of comorbidities	
Median (IQR)	6 (4.25–7.0)
Range	3–10
Types of medical conditions	
Most common types of medical conditions *(does not add up to 20 as patients had* > *1 comorbidity)*	Heart disease – 16 Heart failure – 10 Non-malignant lung disease (e.g., chronic obstructive pulmonary disease [COPD], asthma, pulmonary fibrosis) – 14 Diabetes – 11
Smoking Status, n	
Yes	1
No	7
Previous	12
Living Status, n	
Alone	7
With spouse	11
With ex-spouse	1
Sheltered Living (Alone)	1
Interview with carer, n	**Total carer population, n** = **5**
Yes	5
No	15
Carer relationship to patient	
Spouse (living with patient)	4 (3 Female, 1 Male)
Child (adult daughter, not living with patient)	1 (Female)

^a^mMRC 1 – Breathless when hurrying on the level, or walking up a slight hill.

mMRC 2 – Walks slower than most people on the level, or stop after a mile or so, or stop after 15 min at your own pace.

mMRC 3 – Stops for breath after walking about 100 yards or after a few minutes on level ground.

mMRC 4 – Too breathless to leave the house, or breathless undressing.

**Table 2:** Clinician characteristics.

Age (years)	Total clinician population, n = 10
Median (IQR)	43 (39–48)
Range	32–52
Years of practice	
Median (IQR)	15 (7–22)
Range	4–32
Role	
General Practitioners with Extended Roles (GPwER) in Frailty^a^	4 (Recruited from ICC)
General Practitioner (GP)	1^b^
Advanced Clinical Practitioners (ACPs)	3^b^
Respiratory Practice Nurse	1^b^
Practice Nurse	1^b^

^a^General Practitioners with Extended Roles (GPwER) – A GP who undertakes additional roles beyond the scope of their GP training and which requires supplementary training^52^.

^b^Recruited from one primary care practice.

Four main themes were developed with sub-themes (see Table 3). Application of theoretical framework domains has also been detailed here.

**Table 3:** Themes and Sub-themes.

Qualitative Theme	Sub-themes	Theoretical frameworks and their domains (if applicable)
Theme 1: Widespread Negative Impact of Chronic Breathlessness	—	*Total Dyspnea* • Physical • Psychological • Social *Breathing Space* • Help-seeking behaviours (in crisis only)
Theme 2: Barriers to Optimal Health-Seeking Behaviour and Identification of Chronic Breathlessness	• Experiences of identification/assessment • Experiences of barriers to effective identification of chronic breathlessness • Use of ‘chronic breathlessness’ terminology	*Breathing Space* • Clinicians responsiveness to breathlessness (to breathlessness and underlying disease) • Clinicians responsiveness to breathlessness (to underlying disease only) • Help-seeking behaviours (for persistent breathlessness) • Help-seeking behaviours (in crisis only) • Patients’ coping (disengaged)
Theme 3: Variations in Clinical Management of Chronic Breathlessness	• Variations in treatment and management • Examples of good practice	*Breathing Space* • Clinicians responsiveness to breathlessness (to underlying disease only) • Clinicians responsiveness to breathlessness (to breathlessness and underlying disease) • Patients’ coping (engaged) *Total Dyspnea* • Existential
Theme 4: The Need for Education and Information about Chronic Breathlessness	• Clinicians knowledge and expectations of care regarding chronic breathlessness • Patient’s knowledge and expectations of care regarding chronic breathlessness	*Breathing Space* • Clinicians responsiveness to breathlessness (to breathlessness and underlying disease) • Clinicians responsiveness to breathlessness (to underlying disease only) • Patients’ coping (disengaged)

### Theme 1: Widespread negative impact of chronic breathlessness

Chronic breathlessness has a widespread negative impact on older, frail adults and their carers, adversely affecting psychological wellbeing and quality-of-life. The impact is all-encompassing, affecting all parts of the individual’s life. Patients report depression, anxiety and stress, and carers report feeling frightened, useless, and overwhelmed.

*“Well it sometimes it [the breathlessness] makes me, real down but I don’t try and get, you know what I mean I try and pick myself up and that and think oh well, it’s, it’s the condition, you’ve gotta get on with it so.” (Patient 1, Female, 70 years; Total Dyspnea – psychological)*

*“…..and that’s when I said, what three in the morning it was,…I’m gonna ring an ambulance because she’s gonna, she looks if she’s gonna die she was (inhales sharply) gasping…You know it’s like, at the time of childbirth you’re stuck out of the room when, …you were stuck out the room and, you did the pacing, you know you’re just, useless.”(Carer 2, Husband of Patient 9; Breathing Space - help-seeking behaviours [in crisis only])*

Patients reported how they reduced physical activity and usual activities *due to breathlessness*. Clinicians also identified how patients reduced physical activities, resulting in a downward cycle of deconditioning.

*“…I can’t get about, without help. So it’s, you know it’s extremely…debilitating.” (Patient 3, Female, 69; Total Dyspnea - physical)*

*“Well they just keep they just restrict their life further and further, ‘cause they worry more and more about their breathlessness and…their world kind of shrinks doesn’t it, they used to be able to walk down to the shops, now they can’t so they only get halfway so they don’t bother. And then because they’re not doing anything or exercising they can do less so then they’re stuck in the house. And it’s just a downward spiral basically.” (Clinician 1, GPwER; Total Dyspnea – physical, psychological, social)*

Patients reported a negative social impact resulting from chronic breathlessness, relating to not getting out much on their own, or reduction in hobbies and activities. One participant reflected on how their whole world had transformed.

*“…I’m living in a ‘no world’ now. There’s no decent food, there’s no alcohol, there’s no sex, there’s no driving, you know. Everything is a no.” (Patient 15, Male, 78; Total Dyspnea – physical, psychological, social)*

Despite its widespread negative impact, chronic breathlessness becomes a solitary burden, whereby patients prefer not to share with family/friends, mainly to avoid upsetting others.

*“…our children are in their fifties now. But they’re still our children. And, you know it is silly I know, but, you want to protect them still. So, you don’t tend to tell them about your ailments…it’s not something as a family, we would talk about…unless there was an emergency. And then we would.” (Carer 4, Wife of Patient 15)*

### Theme 2: Barriers to optimal health-seeking behaviour and identification of chronic breathlessness

There were considerable barriers to effective identification of chronic breathlessness, these relate to the experiences of identification/assessment, experiences of barriers to effective identification of chronic breathlessness, and use of ‘chronic breathlessness’ terminology.

#### Experiences of identification/assessment

Various forms of identification and assessment were reported by patients and clinicians, from general observation to detailed assessment (usually for people with chronic obstructive pulmonary disease [COPD] where national criteria apply due to the Quality and Outcomes Framework [QOF] – a financial incentive payment in UK primary care). Some patients reported that they had not had their breathlessness assessed at all. Where breathlessness was assessed, the focus appeared to be primarily in assessing the cause(s) of breathlessness in *disease* terms (e.g., lung function, x-rays), rather than understanding severity/impact of the *symptom* and using this to direct tailored interventions.

*“I literally went for the cough. And I mentioned the breathlessness at the same time you see. And, so she [clinician] sent me for the x-ray.” (Patient 5, Male, 83; Breathing Space – help-seeking behaviours [for persistent breathlessness])*

Although in general clinicians appeared confident that concerns about breathlessness would be raised unprompted by the patient, there were examples of good practice regarding symptom-focussed therapy, such as clinicians asking about breathlessness impact. Two clinicians described how they proactively ask their patients about the impact of breathlessness on their lives.

*Clinician: “-it is the first question I ask, what does it stop you doing? What could you do, before you were breathless that you can’t do now? Erm, and that brings out, a lot of the, the sort of, problems that they’re getting and, and, and how it’s impacted them.” (Clinician 1, GPwER; Breathing Space - clinicians’ responsiveness to breathlessness [responsiveness to breathlessness and underlying disease])*

*Clinician: “Well erm…it depends, most patients…if you start it off openly and they get chatting, within the first couple of minutes they’ve basically told you what they can’t do and what they miss doing…it’s one of those things that impacts so profoundly on their life…it can’t help but come out when you’re asking about how they are and what things have happened…it’s life changing.” (Clinician 3, GPwER; Breathing Space - clinicians’ responsiveness to breathlessness [responsiveness to breathlessness and underlying disease])*

However, in general, patients reported they are *not* asked about breathlessness impact and do not volunteer this information. This leads to a ‘don’t ask, don’t tell’ situation. Two patients report whether their clinician asks about impact of breathlessness.

*Patient 12: “No they, they never do really no.”*

*Patient 11: “No not really no they just seem to do these tests…” (Patient 11, Female, 88 and Patient 12, Male 87 – married couple; Breathing Space - clinicians’ responsiveness to breathlessness [responsiveness to underlying disease only])*

Few clinicians reported using breathlessness measures e.g., the mMRC. Where used, this was predominantly by nurses as part of routine chronic disease management (e.g., COPD annual reviews), however for other causes of breathlessness this did not happen.

*“And I must admit the, MRC breathlessness scale is probably something that I don’t use quite as often as I should do.” (Clinician 5, ACP)*

#### Experiences of barriers to effective identification of chronic breathlessness

Clinicians report breathlessness as a common symptom which is difficult to manage. For the older, frail adult, breathlessness is often one of many symptoms and not always prioritised by them to mention. As a result, chronic breathlessness may only be reported in moments of crisis.

*“I don’t go unless I have to go, unless like, I’m really, out of breath…and I’m wheezing and I’m coughing. That’s when I go. I mean if I’m breathless, I don’t, I don’t bother with them.” (Patient 1, Female, 70; Breathing Space - help-seeking behaviours [in crisis only])*

*“And the thing is as well I think…because of all the, ailments [patient’s] got, I think the breathing, is the bottom of the pile. So, if we can get in to see a doctor, it’s usually for something else.” (Carer 4, Wife of Patient 15; Breathing Space - patients’ coping [disengaged])*

When patients do seek help from primary care, they report difficulty in getting appointments, lack of continuity of care, and lack of time in appointments restricting, either explicitly or implicitly, to ‘one appointment, one problem’. Therefore, if breathlessness is not their most immediately serious symptom, it may not be mentioned *at all*.

*“Nah I mean I think the doctors now they’re that tight for time and, you know you’ve got to be in and out like, it’s like speed dating with the doctor now.” (Carer 2, Husband of Patient 9)*

Clinicians expressed frustration at inadequate length of appointments, particularly for chronic condition management in context of poor GP staffing levels in the area (fewer GPs per head of population than the national average).

*“…a lot of practices have struggled to recruit so a lot of them have emergency care practitioners, which are great – they’re absolutely excellent for an acute breathlessness – but I think some of the chronic disease work is getting missed.” (Clinician 2, GpwER)*

Clinicians report other barriers to reporting breathlessness, such as guilt (relating to smoking). Some patients do not see their primary care clinician for breathlessness *at all*, due to perceived barriers, previous poor experiences, and loss of faith in clinicians for help with this symptom.

*“In fact, unless I’m dying you know unless I’m really ill and then I’d ring 999. I won’t even bother doctor. I’ve no patience nowadays with them.” (Patient 14, Female, 83; Breathing Space - help-seeking behaviours [in crisis only])*

#### Use of ‘chronic breathlessness’ terminology

Patients and carers had limited understanding of ‘chronic breathlessness’ terminology, referring instead to ‘being breathless’. ‘Chronic’ was understood as ‘very severe’ rather than referring to duration, and they did not identify with it, partly because of a sense of inevitability/fatalistic views that nothing could be done to modify breathlessness.

*“Yeah, it’s no good it’s, like you were saying chronic, er, breathless syndrome, you can call it COPD, you know, you can call it what you like, it doesn’t alter the facts of what you’ve got, does it?” (Patient 13, Male, 76)*

Most patients appeared to conflate breathlessness with their condition rather than consider it in any distinct manner.

*“Well it’s got a name hasn’t it, it got COPD, that’s, that is the condition isn’t it, breathlessness.” (Patient 1, Female, 70)*

In contrast, clinicians had a good understanding, and were mainly supportive of the terminology. Some participants suggested that a formal definition of chronic breathlessness syndrome would be useful to raise awareness, create access to services and improved management, and legitimise their symptom as a therapeutic target for identification, assessment and management. However, others were concerned at “labelling” patients and the potential to increase anxiety.

*“But these people have already got a lot of other things going on, so anything that gives it, more visibility is always gonna help.” (Clinician 6, ACP)*

*“But I don’t think then, labelling that as a diagnosis in the, problem pages of patients and then they’re going around labelling that and then telling them that, is is only, only useful in our anxiety, stimulant for the patient.” (HCP 3, GpwER)*

Some clinicians reflected that they found it helpful that other chronic conditions had been defined (e.g., chronic pain, chronic fatigue), focussing on symptom management rather than treatment of the underlying condition.

*“It’s a bit like chronic pain you know…and you can see it, you can measure it, in fact it’s perhaps easier to ascertain than chronic pain ‘cause, you can see it it’s there.” (Clinician 9, ACP)*

### Theme 3: Variations in clinical management of chronic breathlessness

Management of chronic breathlessness was reported as variable by patients and clinicians. This includes variation in techniques/tools for breathlessness management, and examples of good practice.

#### Variations in treatment and management

Management including pharmacological, non-pharmacological, and self-directed strategies were reported by patients and clinicians.

Patients mainly reported receiving pharmacological treatment for the underlying condition e.g., inhaled medication for COPD, or antibiotics/steroids for infective exacerbations; that is, only as indirect management of their breathlessness. Only clinicians discussed the potential use of opioids as a pharmacological intervention for breathlessness itself.

*“No, only only me nebulator [nebuliser] but I I don’t use that often…but er…I always have me pump [inhaler], I’ve had one in every jacket pocket.” (Patient 13, Male, 76; Breathing Space - clinicians’ responsiveness to breathlessness [responsiveness to underlying disease only])*

*“And in terms of maximal optimal treatment I suppose if they haven’t, er if that doesn’t include opiates erm I’d use that as well.” (Clinician 3, GPwER)*

Clinicians reported use of non-pharmacological breathlessness management options, but these were less often mentioned by patients. These included breathing exercises, calming hand, fan, relaxation, rest, and referrals (e.g., physiotherapy, COPD/pulmonary rehabilitation, or breathlessness clinics). Most clinicians referred to the management of patients within the context of COPD.

*“…there’s the non-pharmacological stuff basically to pulmonary rehab, breathing exercises, er CBT [cognitive behavioural therapy], you know using a fan on the face that sort er, getting people to pace themselves better erm, I suppose anxiety management’s a lot of it too…” (Clinician 1, GPwER; Breathing Space - clinicians’ responsiveness to breathlessness [responsiveness to breathlessness and underlying disease])*

Some patients reported how they adapted their behaviour and developed their own self-management strategies which included resting, breathing exercises, mobility aids (electric scooters, walkers), exercise, thinking positively, or inhalers/nebulisers. One patient referred to breathing techniques taught by physiotherapists as being very useful. Religious beliefs and faith/spirituality were also described by some as a coping strategy.

*“Well as I say I go out on my scooter I’m able to do my own shopping…when I first got the scooters the boys said ‘Oh Mam you’re giving up’. But I said ‘No on the contrary it’s what’s gonna keep me going’.” (Patient 4, Female, 70; Breathing Space – patients’ coping [engaged])*

*“You’ve gotta have, beliefs. I said it’s no good having my illnesses, and not believing that…I’ve got longer. I said I aren’t give up on life yet.” (Patient 18, Female, 81; Total Dyspnea – existential; Breathing Space - patients’ coping [engaged])*

Carers were integral to management, providing medical/social support to help the patient with their breathlessness e.g., cooking, driving, shopping, attending/keeping records of appointments, and understanding the patients’ care needs.

#### Examples of good practice

There were examples of good practice from clinicians using a holistic patient-centred approach even though the limits of disease-directed treatment had been reached.

*“…I don’t effectively do anything, it’s just that chat that gets him through. It’s quite frustrating ‘cause I can’t manage him with anything ‘cause he’s intolerant of everything. Er, and he’s been through the lot so the only support I can give him is just to, have a chat.” (Clinician 3, GPwER; Breathing Space - clinicians’ responsiveness to breathlessness [responsiveness to breathlessness and underlying disease])*

A few clinicians used outcome measures to help assess breathlessness, and most used general observation e.g., when entering the treatment room. Regular follow-up appointments, practice-based group consultations where patients can learn from each other, providing education/information about symptom, condition, and medications to patients/carers, and practitioner peer support were also reported.

*“Erm, I’ve recently set up like group consultations erm, here for COPD. Erm, because I think again, these are a group that, benefit hugely by others, erm and support because quite often, isolation and depression is part of, COPD, because they can’t get out maybe as much or, they’re erm breathless and they don’t want to, you know erm, exert their selves.” (Clinician 8, Respiratory Practice Nurse; Breathing Space - clinicians’ responsiveness to breathlessness [responsiveness to breathlessness and underlying disease])*

*Clinician: “I think [patient] education is just massive. It’s education all the time.” (Clinician 10, Practice Nurse)*

### Theme 4: The need for education and information about chronic breathlessness

This theme described the differences in knowledge, management, and expectations of care regarding chronic breathlessness, from the patient, carer, and clinician perspective.

#### Clinicians’ knowledge and expectations of care regarding chronic breathlessness

In general, clinicians appeared to have a good knowledge, awareness, and understanding of breathlessness and its impact on both patient and carers.

*“Yeah I think I’ve understood a little bit more about, about the symptom rather than the pathology behind it.” (Clinician 1, GPwER, after attending a palliative care course about breathlessness; Breathing Space - clinicians’ responsiveness to breathlessness [responsiveness to breathlessness and underlying disease])*

However, even when they demonstrated good knowledge or confidence about the symptom and its causal diseases, clinicians still reported feeling ‘helpless’ to manage breathlessness.

*“…understanding as a symptom and, is different to our understanding of the pathology behind it. Erm I think, as doctors we’re all, quite smug about understanding the pathology but, but that doesn’t always mean we can treat the symptom, and we’re often quite useless at treating the symptom, even though we understand what’s going on (laughs).” (Clinician 1, GPwER; Breathing Space - clinicians’ responsiveness to breathlessness [responsiveness to underlying disease only])*

#### Patient’s knowledge and expectations of care regarding chronic breathlessness

Patients and carers demonstrated poor knowledge of, and had low expectations about, breathlessness-directed treatments.

*Carer: “Well yeah you take you’re taking your inhalers.”*

*Patient: “That’s all. Yes.”*

*Carer: “Well that’s all there is isn’t it.” (Patient 15, Male, 78 and Carer 4, Wife of Patient 15; Breathing Space – patients’ coping [disengaged])*

If considered specifically, breathlessness was under-reported, normalised, and attributed to growing older.

*“I never spoke about it when I first had it. I never bothered. I just thought it was old age.” (Patient 8, Male, 92)*

Patients lack of consideration of breathlessness as something to report to their clinician was compounded by the perceived lack of time, and disease-focused nature of primary care contacts. This was not conducive to being educated about breathlessness and its management although the importance of education was recognised by one patient.

## Discussion

Chronic breathlessness seriously impacts frail, older adults and their carers, reducing physical and psychological wellbeing and quality-of-life. Frail older adults saw their breathlessness as one symptom among many which was mostly not prioritised during clinician consultations. Primary care consultations were viewed as difficult to access, with time only for the most pressing problem perceived as most amenable to treatment, and lacked continuity. Patients assumed their breathlessness was inevitable, synonymous with their disease and, as clinicians did not usually initiate discussion or offer specific interventions, had no effective management other than treatment of the causative disease. This nihilism fed the non-prioritisation and a “don’t ask, don’t tell” situation. Patients, therefore, did not recognise “chronic breathlessness” as a specific entity, legitimate to bring to their clinicians with specific treatments to help them live more easily. The term “chronic” was misinterpreted by patients as meaning “severe” rather than “persistent” or “long-term”. Clinicians, whilst appreciating the impact on patients’ lives, mostly felt powerless to help breathlessness. Given past experiences of people with severe, frightening breathlessness volunteering their concerns, clinicians assumed that patients would tell them if it remained bothersome without routine enquiry. Most clinicians felt that delineating “chronic breathlessness” as a specific entity was useful to encourage identification and management, although some were concerned about “labelling” patients. However, despite the communication impasse rendering breathlessness invisible, there were examples of excellent and innovative practice whereby clinicians initiated proactive care for people with chronic breathlessness.

Our findings are consistent with literature pertaining to the general adult population showing chronic breathlessness is associated with worse physical and mental QoL^5^ and restrictions of activities^6^. The all-pervading experience of breathlessness illustrates the Total Dyspnoea concept whereby breathlessness is experienced throughout all domains of life^36^. This concept was developed in order to understand the psychological, physical, social, and existential/spiritual experiences of chronic breathlessness^36^ and has good explanatory value for our findings. In older adults, lack of activities and restricted mobility can decrease functional performance and result in deconditioning, leading to social withdrawal and a downward spiral of frustration, loneliness, and depression^39^, with increased risk of hospital admission^40^. Anxiety and depression were associated with restricting breathlessness in a cohort of older adults (≥70)^4^. Our findings also confirm previous literature showing the caregiver burden of those supporting individuals with chronic breathlessness^9,10^. The impact of frailty and the presence of other long-term conditions are additional factors to consider.

The importance of clinician-focus on breathlessness as well as the disease, and appropriate help-seeking by the patient are key aspects of the Breathing Space framework which facilitate the patient with breathlessness to live as well as possible^1^. The Breathing Space framework considers patient coping (engaged/disengaged), help-seeking behaviours (for persistent breathlessness/in crisis only), and clinicians’ responsiveness to breathlessness (clinician responsive to breathlessness and underlying disease/clinician responsiveness to disease only^1^) on quality of life, and also showed good explanatory value for our findings. However, despite instances of good practice described by the clinician participants, for frail older adults living with a number of long-term conditions, a model of primary care with short, pressured and difficult to obtain appointments, with different clinicians does not support the presentation of chronic breathlessness as a legitimate concern. The model of primary care based on ten-minute appointments has been highlighted as no longer fit for purpose due to several contributing factors, including an ageing population with multiple comorbidities^41^. This, and an assumption that no breathlessness-directed treatment is possible leads to a communication impasse between patient and clinician. Although the invisibility of^23,24,42^, and therapeutic nihilism about, breathlessness has previously been reported, our findings highlight the additional challenges for older adults with multi-morbidity leaving some to seek help only during crisis.

Notwithstanding good practice examples, our data confirm previous reports of a lack of systematic identification and assessment of breathlessness in primary care settings other than as part of COPD chronic disease management^43^. However, chronic disease management models in primary care such as those supported by the Quality and Outcomes Framework in the UK^44^ may provide a useful framework to support good breathlessness identification and assessment in all patients with conditions which cause breathlessness. This in turn may provide better management for this debilitating symptom.

The consensus process to define “chronic breathlessness syndrome”^25^ did not include patient or carer views. Our findings show that patients have such a major lack of understanding of *chronic breathlessness* as a specific entity with effective treatments, that the term was meaningless. In addition, the term “chronic” was misunderstood as “severe” and rejected as applying to themselves. Therefore, education is vital for patients to understand the concept of chronic breathlessness as a legitimate target for treatment. The term “persistent” or “long-term” may be better than “chronic”. Clinicians, for the most part, felt that having a defined terminology is useful in raising awareness, and encouraging identification and treatment. For the patient, even the first step in appropriate help-seeking – that is, recognising that chronic breathlessness as a distinct and legitimate entity to report to the GP - is blocked if they believe “nothing can be done” (due to normalisation of breathlessness/adjusting their lives to minimise impact^22^) and that breathlessness is synonymous with their disease(s).

Despite an increasing evidence base for breathlessness-directed interventions^45–48^ including those helping to promote self-management^45^ and engagement with ways to maximise living well - another key aspect of the Breathing Space concept^1^ - few patients reported receiving *any*, other than those few with good, engaged coping had found for themselves. Although clinicians showed good, general knowledge *about* breathlessness, they appeared to have poor knowledge and confidence about its management, often feeling helpless and powerless in the face of this symptom consistent with the published literature^49,50^.

### Strengths and limitations

We were able to collect rich data from a purposively selected sample of patients. Including views of clinicians as well as patients and carers provided a broader view of the issue.

The patient and clinician interviews were not linked which may explain the disparity in accounts regarding management of breathlessness, however this provided opportunity to show contrasting views. The clinician participants were highly motivated and had additional expertise regarding older adults with frailty or worked in the same practice as those that did. Clinicians described excellent person-centred holistic practice and may not be a representative of all primary care clinicians.

Finally, this study was conducted in a deprived area with one of the highest prevalence of respiratory disease in England and findings reflect this context^51^. However, whilst qualitative work does not purport to provide generalisable data, findings are likely to be applicable to other geographical areas.

### Implications for research and practice

Chronic breathlessness in the older, frail adult in primary care is a neglected issue with widespread negative impact on people living with breathlessness and their carers. To better understand the experience of this population, and to allow patients to report their symptoms in the clinical environment, *systematic enquiry through routine identification and assessment of chronic breathlessness, initiated by the clinician, should be adopted.* Frail older adults are likely to have chronic breathlessness but unlikely to report it to their primary healthcare clinicians. The Electronic Frailty Index, or other tools are increasingly used to identify people at risk of frailty in general practice. Identification of people at risk of frailty using the EFI should prompt enquiry about the presence and impact of breathlessness.

Asking about experiences of breathlessness alongside measurement would open discussion with patients and help tailor management. This could include pharmacological and non-pharmacological treatments delivered by primary care clinicians. Urgent review of the ‘one appointment, one problem’ model of care for older adults with multiple long-term conditions is needed. Additionally, education about breathlessness, its treatment, and management, would be beneficial for patients, carers, and clinicians.

Future research should recognise that many of the studies forming the evidence base for breathlessness management should include older adults, and those with frailty and multiple long-term conditions. Further work to adapt the current definition of chronic breathlessness to give patient-relevant terminology - such as ‘persistent breathlessness’ – could benefit future patient/carer understanding of breathlessness, potentially preventing conflation between breathlessness and underlying disease(s). Research addressing whether interventions not only reduce breathlessness severity but also facilitate a return to activities, or maintenance of independent home-living, along with consideration of measuring outcomes which are the most meaningful to individuals, would be beneficial to the older, frail population.

## Conclusion

This study adds new evidence to the well-established literature base regarding the burden and widespread negative impact of chronic breathlessness, particularly, on the *older, frail adult* (and their carer); evidence about chronic breathlessness in this population is scarce.

Chronic breathlessness is distressing to the older, frail adult (and their carer), with significant negative impacts on psychological wellbeing and QoL. Barriers to adequate identification and assessment of this symptom within the primary care setting are evident. Mixed views of chronic breathlessness syndrome terminology demonstrated by patients, exemplified their lack of understanding or recognition of breathlessness as a symptom with therapeutic target.

Differences in opinions about treatment and management of breathlessness from patients/carers and clinicians (despite some examples of good practice on the part of the clinician), coupled with poor knowledge and understanding from *both* patient and practitioner, sustain the concept of the invisibility of breathlessness. This population may be particularly invisible when chronic breathlessness is one of many symptoms. Consequently, breathlessness remains *unidentified and untreated*, with adverse impacts on the older, frail adult.

## Data Availability

The anonymised data underlying this article will be made available on request by authorised researchers via https://hull-repository.worktribe.com/output/4719318, following completion of a data sharing agreement. To request access, contact worktribe@hull.ac.uk quoting the Worktribe output number (4719318).

## Appendix A - Patient/Carer Topic Guide

*Questions*

PSYCHOLOGICAL IMPACT OF BREATHLESSNESS

1. Can you tell me a bit about your <<or name’s > > breathlessness?
  - How long?
  - Impact on daily life?
  - How does it make you feel in yourself?

ENCOUNTER WITH DOCTOR/NURSE ETC

2. Let’s think about if you <<they > > visit or contact your GP surgery about your breathlessness. Can you tell me about this? Who? (who brings up topic of breathlessness)
  - What was it like getting an appointment? (Did you say why you needed an appointment?)
  - What was discussed about your <<their > > breathlessness?
  - How well do you think they see or understand your breathlessness?
  - Do you feel happy with the treatment/help they give you (how they deal with the problem)?
  - How does this make you feel?
3. How easy or otherwise do you find it to talk about your <<their > > breathlessness with your GP, nurse, or other health professional and why? (How do you feel in the appointment?)
  - How easy was it to talk about when it first appeared/or if it persists despite treatment
  - Disease/cause vs symptom
  - Interested, not interested in symptom
  - Legitimacy of taking symptom to GP practice
  - Barriers (difficulties) and facilitators (help) in mentioning/discussing breathlessness
  - How does that make you feel? (e.g., empowered, listened to, frustrated, resigned, helpless etc)
4. What is important/helpful to you in the way your <<their > > GP, nurse, or other health professional listens to you about your breathlessness and the things they might do to help?
  - How does GP/HCP respond?
  - Is the GP/HCP responsive/engaged/disengaged? (what about in the long term?)
  - Barriers (difficulties) and facilitators (help) in mentioning/discussing breathlessness
  CHRONIC BREATHLESSNESS DEFINITION
5. Do you think having a name for your daily breathlessness, such as chronic breathlessness, would help make it easier to understand? What are your thoughts about that?
  - Would it be helpful to say you have chronic breathlessness, in addition to COPD, Heart Failure etc?
  - How might a name make it more real/legitimate/visible (e.g., help you to talk to your GP team, help them to ask you about it)?
  - How would you feel about this?
6. Is there anything else (patient and/or carer) that you would like to say or discuss?
  Those not seeing Primary Care Health Care Practitioner
  - Why do you choose not to see a primary care health care practitioner?
  - What would make you go to a primary care health care practitioner?
  - Who do you speak to instead?

## Appendix B – Health Care Practitioner Topic Guide

*Questions*

1. Can you tell me about your experiences when presented with someone suffering with chronic breathlessness?
  - What happens/what was discussed? Who raises the topic of breathlessness/how do you determine they are breathless?
  - How easy do you think it is for them (or you) to raise the topic of breathlessness?
  - How do you think chronic breathlessness affects those that you see (patients and carers?)
  - Do you use any outcome measurements for breathlessness, e.g., mMRC, VAS, NRS
    1. If no, do you think they would useful (changes over time etc)?
2. How do you feel when presented with someone with chronic breathlessness and any associated side effects (physical or psychological)?
  - Adequately prepared, comfortable, well equipped, not prepared
  - Do you ask about how their breathlessness impacts their life?
3. What are your thoughts about recognising chronic breathlessness as a syndrome in its own right?
  - How might a name make it more real/legitimate/visible (e.g., increase awareness)?
  - What does it mean to you as a practitioner?
  - Designation of syndrome raise awareness amongst colleagues?
4. How do you feel about telling someone they have chronic breathlessness syndrome?
5. Is there anything else that you would like to say or discuss about chronic breathlessness (about practitioners, patients, carers, or the primary care encounter)?

## References

[1] Hutchinson, A., Barclay-Klingle, N., Galvin, K. & Johnson, M. J. Living with breathlessness: a systematic literature review and qualitative synthesis. *Eur. Respir. J.* **51**, 1701477 (2018). 29467199 10.1183/13993003.01477-2017

[2] Ekström, M. P., Abernethy, A. P. & Currow, D. C. The management of chronic breathlessness in patients with advanced and terminal illness.*BMJ* **349**, g7617 (2015). 10.1136/bmj.g7617 25556037

[3] Janssen, D. J. A., Wouters, E. F. M. & Spruit, M. A. Psychosocial consequences of living with breathlessness due to advanced disease. *Curr. Opin. Support. Palliat. Care* **9**, 232–237 (2015). 26125305 10.1097/SPC.0000000000000146

[4] Johnson, M. J. et al. Breathlessness in elderly adults during the last year of life sufficient to restrict activity: prevalence, pattern, and associated factors. *J. Am. Geriatr. Soc.* **64**, 73–80 (2016). 26782854 10.1111/jgs.13865 PMC4719155

[5] Currow, D. et al. Chronic breathlessness associated with poorer physical and mental health-related quality of life (SF-12) across all adult age groups. *Thorax* **72**, 1151–1153 (2017). 28356419 10.1136/thoraxjnl-2016-209908

[6] Kochovska, S. et al. Activities Forgone because of chronic breathlessness: a cross-sectional population prevalence study. *Palliat. Med. Rep.* **1**, 166–170 (2020). 34223472 10.1089/pmr.2020.0083 PMC8241375

[7] Hutchinson, A., Pickering, A., Williams, P., Bland, J. M. & Johnson, M. J. Breathlessness and presentation to the emergency department: a survey and clinical record review. *BMC Pulm. Med.* **17**, 53–59 (2017). 28320369 10.1186/s12890-017-0396-4 PMC5360046

[8] Currow, D. C. et al. Health service utilisation associated with chronic breathlessness: random population sample. *ERJ Open Res.* **7**, 00415–02021 (2021). 34651042 10.1183/23120541.00415-2021 PMC8503326

[9] Booth, S., Silvester, S. & Todd, C. Breathlessness in cancer and chronic obstructive pulmonary disease: using a qualitative approach to describe the experience of patients and carers. *Palliat. Support. Care* **1**, 337–344 (2003). 16594223 10.1017/s1478951503030499

[10] Simpson, A. C., Young, J., Donahue, M. & Rocker, G. A day at a time: caregiving on the edge in advanced COPD. *Int. J. Chronic Obstr. Pulm. Dis.* **5**, 141–151 (2010). 10.2147/copd.s9881 PMC2898087 20631814

[11] Ferreira, D. H., Kochovska, S., Honson, A., Phillips, J. L. & Currow, D. C. Two faces of the same coin: a qualitative study of patients’ and carers’ coexistence with chronic breathlessness associated with chronic obstructive pulmonary disease (COPD). *BMC Palliat. Care* **19**, 1–12 (2020). 32375747 10.1186/s12904-020-00572-7 PMC7203967

[12] Lund, L., Ross, L., Petersen, M. A. & Groenvold, M. The interaction between informal cancer caregivers and health care professionals: a survey of caregivers’ experiences of problems and unmet needs. *Support. Care Cancer* **23**, 1719–1733 (2015). 25432867 10.1007/s00520-014-2529-0

[13] Johnson, M. J., Currow, D. C. & Booth, S. Prevalence and assessment of breathlessness in the clinical setting. *Expert Rev. Respir. Med.* **8**, 151–161 (2014). 24450289 10.1586/17476348.2014.879530

[14] Currow, D. C., Plummer, J. L., Crockett, A. & Abernethy, A. P. A community population survey of prevalence and severity of dyspnea in adults. *J. Pain. Symptom Manag.* **38**, 533–545 (2009). 10.1016/j.jpainsymman.2009.01.006 19822276

[15] Baxter, N. Breathlessness in the primary care setting. *Curr. Opin. Support. Palliat. Care* **11**, 152–158 (2017). 28644300 10.1097/SPC.0000000000000284

[16] Moens, K., Higginson, I. J. & Harding, R. Are there differences in the prevalence of palliative care-related problems in people living with advanced cancer and eight non-cancer conditions? A systematic review. *J. Pain. Symptom Manag.* **48**, 660–677 (2014). 10.1016/j.jpainsymman.2013.11.009 24801658

[17] Berliner, D., Schneider, N., Welte, T. & Bauersachs, J. The differential diagnosis of dyspnea. *Dtsch. Arzteblatt Int.* **113**, 834–845 (2016). 10.3238/arztebl.2016.0834 PMC5247680 28098068

[18] Booth, S., Bausewein, C., Higginson, I. & Moosavi, S. H. Pharmacological treatment of refractory breathlessness. *Expert Rev. Respir. Med.* **3**, 21–36 (2009). 20477280 10.1586/17476348.3.1.21

[19] Brighton, L. J. et al. Holistic services for people with advanced disease and chronic breathlessness: a systematic review and meta-analysis. *Thorax* **74**, 270–281 (2019). 30498004 10.1136/thoraxjnl-2018-211589 PMC6467249

[20] Bausewein, C. et al. Breathlessness services as a new model of support for patients with respiratory disease. *Chronic Respir. Dis*. **15**, 48–59 (2018). 10.1177/1479972317721557 PMC5802660 28718321

[21] Lorig, K. R. & Holman, H. R. Self-management education: history, definition, outcomes, and mechanisms. *Ann. Behav. Med.* **26**, 1–7 (2003). 12867348 10.1207/S15324796ABM2601_01

[22] Kochovska, S. et al. Invisibility of breathlessness in clinical consultations: a cross-sectional, national online survey. *Eur. Respir. J.* **60**, 2201603 (2022). 36202418 10.1183/13993003.01603-2022

[23] Carel, H., Macnaughton, J. & Dodd, J. Invisible suffering: breathlessness in and beyond the clinic. *Lancet Respir. Med.* **3**, 278–279 (2015). 25890648 10.1016/S2213-2600(15)00115-0

[24] Gysels, M. & Higginson, I. J. Access to services for patients with chronic obstructive pulmonary disease: the invisibility of breathlessness. *J. Pain. Symptom Manag.* **36**, 451–460 (2008). 10.1016/j.jpainsymman.2007.11.008 18495412

[25] Johnson, M. J. et al. Towards an expert consensus to delineate a clinical syndrome of chronic breathlessness. *Eur. Respir. J.* **49**, 1–8 (2017). 10.1183/13993003.02277-2016 28546269

[26] Conroy, S. & Elliott, A. The frailty syndrome. *Medicine* **45**, 15–18 (2017).

[27] Fried, L. P. et al. Frailty in older adults: evidence for a phenotype. *J. Gerontol. Ser. A Biol. Sci. Med. Sci.* **56**, M146–M156 (2001). 11253156 10.1093/gerona/56.3.m146

[28] Ahmedzai, S. H. Breathlessness in advanced disease. *Medicine* **48**, 23–28 (2020).

[29] Smith, A. K. et al. Prevalence and outcomes of breathlessness in older adults: a national population study. *J. Am. Geriatr. Soc.* **64**, 2035–2041 (2016). 27603500 10.1111/jgs.14313

[30] Johnson M. J. et al*.* Breathlessness limiting exertion in very old adults: findings from the Newcastle 85+ Study. *Age Ageing* **52**, afad155 (2023). 10.1093/ageing/afad155 PMC10474592 37658750

[31] Scotland, J. Exploring the philosophical underpinnings of research: Relating ontology and epistemology to the methodology and methods of the scientific, interpretive, and critical research paradigms. *Engl. Lang. Teach.* **5**, 9 (2012).

[32] Murtagh, F. E. et al. A non-randomised controlled study to assess the effectiveness of a new proactive multidisciplinary care intervention for older people living with frailty. *BMC Geriatr.* **23**, 6 (2023). 36604609 10.1186/s12877-023-03727-2 PMC9813451

[33] Malterud, K., Siersma, V. D. & Guassora, A. D. Sample size in qualitative interview studies: guided by information power. *Qua. Health Res.* **26**, 1753–1760 (2016). 10.1177/1049732315617444 26613970

[34] Braun, V. & Clarke, V. Using thematic analysis in psychology. *Qual. Res. Psychol.* **3**, 77–101 (2006).

[35] Braun, V. & Clarke, V. Reflecting on reflexive thematic analysis. *Qual. Res. Sport Exerc. Health* **11**, 589–597 (2019).

[36] Abernethy, A. P. & Wheeler, J. L. Total dyspnoea. *Curr. Opin. Supportive Palliat. Care* **2**, 110–113 (2008). 10.1097/SPC.0b013e328300cad0 18685406

[37] Lincoln Y. S., Guba E. G. Naturalistic inquiry: Sage (1985).

[38] Saunders C. Care of patients suffering from terminal illness at St. Joseph’s Hospice, Hackney, London. Nursing Mirror. **14** (1964).

[39] Parshall, M. B. et al. An official American Thoracic Society statement: update on the mechanisms, assessment, and management of dyspnea. *Am. J. Respir. Crit. Care Med.* **185**, 435–452 (2012). 22336677 10.1164/rccm.201111-2042ST PMC5448624

[40] Bu, F., Philip, K. & Fancourt, D. Social isolation and loneliness as risk factors for hospital admissions for respiratory disease among older adults. *Thorax* **75**, 597–599 (2020). 32317268 10.1136/thoraxjnl-2019-214445 PMC7361022

[41] Flaxman, P. The 10-minute appointment. *Br. J. Gen. Pract.* **65**, 573–574 (2015). 26500303 10.3399/bjgp15X687313 PMC4617250

[42] Currow, D. C. & Johnson, M. J. Chronic breathlessness: silent and deadly. *Curr. Opin. Supportive Palliat. Care* **10**, 221–222 (2016). 10.1097/SPC.0000000000000231 27359078

[43] Elliott-Button, H. L., Johnson, M. J., Nwulu, U. & Clark, J. Identification and assessment of breathlessness in clinical practice: a systematic review and narrative synthesis. *J. Pain. Symptom Manag.* **59**, 724–733 (2019). 10.1016/j.jpainsymman.2019.10.014 31655187

[44] NHS England. A five-year framework for GP contract reform to implement The NHS Long Term Plan. England 2019 [Available from: https://www.england.nhs.uk/publication/gp-contract-five-year-framework/.

[45] Spathis, A. et al. The breathing, thinking, functioning clinical model: a proposal to facilitate evidence-based breathlessness management in chronic respiratory disease. *NPJ Prim. Care Respir. Med.* **27**, 1–6 (2017). 28432286 10.1038/s41533-017-0024-z PMC5435098

[46] Luckett, T. et al. Contributions of a hand-held fan to self-management of chronic breathlessness. *Eur. Respir. J.* **50**, 1700262 (2017). 28818884 10.1183/13993003.00262-2017

[47] Johnson, M. J. et al. A randomised controlled trial of three or one breathing technique training sessions for breathlessness in people with malignant lung disease. *BMC Med.* **13**, 213 (2015). 26345362 10.1186/s12916-015-0453-x PMC4562360

[48] Spathis, A. et al. Cutting through complexity: the breathing, thinking, functioning clinical model is an educational tool that facilitates chronic breathlessness management. *NPJ Prim. Care Respir. Med.* **31**, 1–3 (2021). 33972569 10.1038/s41533-021-00237-9 PMC8110567

[49] Politis, J. et al. Managing severe chronic breathlessness in chronic obstructive pulmonary disease is challenging for general practitioners. *Am. J. Hosp. Palliat. Med.* **38**, 472–479 (2021). 10.1177/1049909120959061 32940530

[50] Lunn, S., Dharmagunawardena, R., Lander, M. & Sweeney, J. It’s hard to talk about breathlessness: a unique insight from respiratory trainees. *Clin. Med.* **19**, 344–347 (2019). 10.7861/clinmedicine.19-4-344 PMC6752249 31308122

[51] MHCLG. The English Indices of Deprivation 2019 (IoD2019): Ministry of Housing, Communities & Local Government; 2019 [Available from: https://assets.publishing.service.gov.uk/government/uploads/system/uploads/attachment_data/file/835115/IoD2019_Statistical_Release.pdf.

[52] Royal College of General Practitioners. General Practitioners with Extended Roles; 2022 [Available from: https://www.rcgp.org.uk/your-career/gp-extended-roles.

